# Anodic electro-fermentation of 3-hydroxypropionic acid from glycerol by recombinant *Klebsiella pneumoniae* L17 in a bioelectrochemical system

**DOI:** 10.1186/s13068-017-0886-x

**Published:** 2017-08-17

**Authors:** Changman Kim, Mi Yeon Kim, Iain Michie, Byong-Hun Jeon, Giuliano C. Premier, Sunghoon Park, Jung Rae Kim

**Affiliations:** 1 School of Chemical and Biomolecular Engineering, Pusan National University, Busan, 609-735 Republic of Korea; 2 Sustainable Environment Research Centre (SERC), Faculty of Computing, Engineering and Science, University of South Wales, Pontypridd, Mid-Glamorgan CF37 1DL UK; 3 Department of Natural Resources and Environmental Engineering, Hanyang University, Seoul, 133-791 Republic of Korea

**Keywords:** 3-hydroxypropionic acid, Electro-fermentation, Bioelectrochemical system, *Klebsiella pneumoniae* L17

## Abstract

**Background:**

3-Hydroxypropionic acid (3-HP) is an important platform chemical which can be produced biologically from glycerol. *Klebsiella pneumoniae* is an ideal biocatalyst for 3-HP because it can grow well on glycerol and naturally synthesize the essential coenzyme B_12_. On the other hand, if higher yields and titers of 3-HP are to be achieved, the sustained regeneration of NAD^+^ under anaerobic conditions, where coenzyme B_12_ is synthesized sustainably, is required.

**Results:**

In this study, recombinant *K. pneumoniae* L17 overexpressing aldehyde dehydrogenase (AldH) was developed and cultured in a bioelectrochemical system (BES) with the application of an electrical potential to the anode using a chronoamperometric method (+0.5 V vs. Ag/AgCl). The BES operation resulted in 1.7-fold enhancement of 3-HP production compared to the control without the applied potential. The intracellular NADH/NAD^+^ ratio was significantly lower when the L17 cells were grown under an electric potential. The interaction between the electrode and overexpressed AldH was enhanced by electron shuttling mediated by HNQ (2-hydroxy-1,4-naphthoquinone).

**Conclusions:**

Enhanced 3-HP production by the BES was achieved using recombinant *K. pneumoniae* L17. The quinone-based electron transference between the electrode and L17 was investigated by respiratory uncoupler experiments. This study provides a novel strategy to control the intracellular redox states to enhance the yield and titer of 3-HP production as well as other bioconversion processes.

**Electronic supplementary material:**

The online version of this article (doi:10.1186/s13068-017-0886-x) contains supplementary material, which is available to authorized users.

## Background

3-hydroxypropionic acid (3-HP; C_3_H_6_O_3_) has attracted interest because of its wide applications in the synthesis of various value-added chemicals, such as acrylic acid, acrylamide, and propiolactone [1, 2]. According to a report by the U.S. Department of Energy, 3-HP is one of the top 12 value-added platform chemicals that can be produced biologically but the process requires urgent development [3]. To date, many studies including the development of genetically and metabolically engineered microbial strains have been conducted to improve the yield and titer for the commercial production of 3-HP [4–7]. 3-HP can be produced from glucose and glycerol as the carbon sources, both of which are renewable and abundant.

**Fig. 1:**
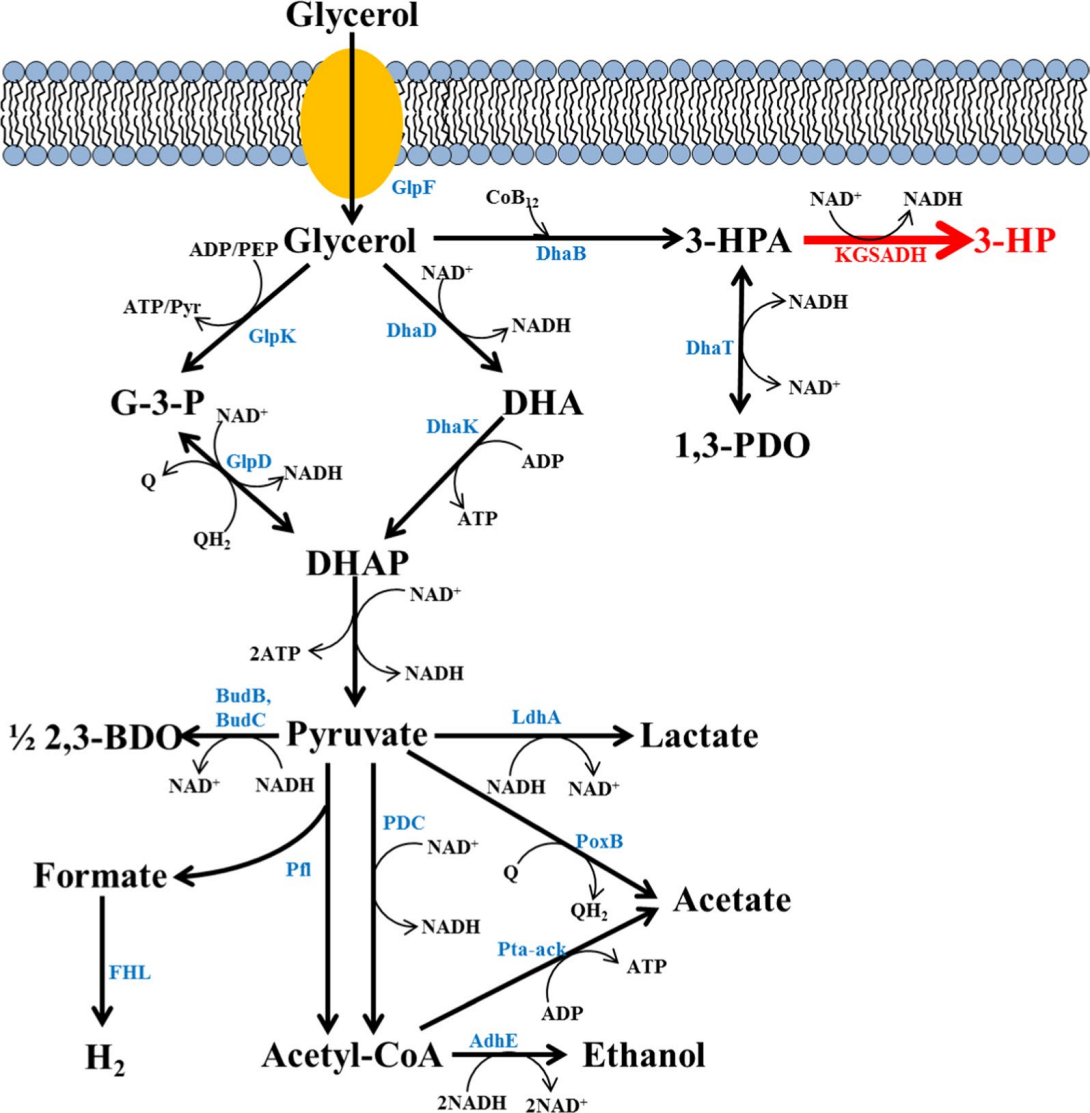
Central glycerol pathway of recombinant *K. pneumoniae* L17

3-HP can be synthesized from glucose as the carbon source via phosphoenolpyruvate or pyruvate. On the other hand, various problems of these pathways, such as the excessive accumulation of toxic byproducts, inappropriate redox balance, insufficient expression of active enzyme(s), and/or negative net ATP generation, have been reported [8]. With glycerol is used as the carbon source, 3-HP is produced by a two-step reaction: glycerol to 3-hydroxypropionaldehyde (3-HPA) by glycerol dehydratase (GDHt) and then to 3-HP by aldehyde dehydrogenase (AldH) (Fig. 1). GDHt essentially requires coenzyme B_12_ or *S*-adenosyl methionine (SAM) as a cofactor, and AldH requires NAD(P)^+^ as a cofactor for 3-HP production. The pathway for glycerol to 3-HP is simpler than that starting from glucose. Furthermore, glycerol is cheaper than glucose because the former is generated abundantly as a major byproduct in the biodiesel industry.

*Klebsiella pneumoniae* is a natural 3-HP producer with several advantages in 3-HP production over other strains. The strain can grow well on glycerol to a high cell density at a fast rate even under anaerobic condition. In addition, it expresses a coenzyme B_12_-dependent GDHt (called DhaB) that is less sensitive to oxygen than the SAM-dependent GDHt. Furthermore, it can produce sufficient amounts of coenzyme B_12_ for the catalysis of DhaB under anaerobic conditions [9]. In comparison, most well-known fermentative hosts, such as *E. coli* do not grow well on glycerol under anaerobic conditions and cannot produce coenzyme B_12_. Coenzyme B_12_ is very expensive ($531/g), making it unsuitable for addition directly to the culture medium. On the other hand, *K. pneumoniae* was reported to produce up to 83.8 g/L of 3-HP without the external supplementation of coenzyme B_12_, which provides a great advantage of mass production of 3-HP [5]. For the successful production of 3-HP by *K. pneumoniae*, the overexpression and/or heterologous expression of highly active AldH is essential due to the low innate AldH activity in the wild-type strains [10].

The reaction by AldH generates NADH from NAD^+^ by the oxidation of 3-HPA to 3-HP; consequently, the continuous regeneration of NAD^+^ is crucial, as shown in Fig. 1. If NAD^+^ is not regenerated properly, this reaction would stop immediately. Furthermore, a high cellular NADH level due to its improper oxidation to NAD^+^ causes the accumulation of undesired byproducts, such as lactate and ethanol. The simplest and most inexpensive way for NAD^+^ regeneration is to accelerate the electron transport chain (ETC). However, NAD^+^ regeneration via aerobic respiration is not preferred when using *K. pneumoniae* as a host for 3-HP production from glycerol. Under aerobic conditions, expression of the DHA regulon, which includes *dhaB*, and the coenzyme B_12_ synthetic pathway are significantly suppressed. In addition, the DhaB enzyme and its cofactor, coenzyme B_12_, are unstable under aerobic conditions [11, 12]. To solve this problem, anaerobic respiration using a chemical electron acceptor, such as nitrate, has been attempted, but it is expensive and the reduction of nitrate yields nitrite, which is toxic and seriously inhibits the cellular metabolism [9, 13].

Some microorganisms, called exoelectrogens, can deliver respiratory electrons to solid electrodes under anaerobic conditions. The microbe–electrode interaction via extracellular electron transfer to an electrode is intriguing because it offers an alternative way to control the cellular redox states, and does not rely on ETC or fermentation (e.g., production of reduced metabolites) [14]. The use of a bioelectrochemical system enables thermodynamically unfeasible metabolisms, i.e., unbalanced fermentation, to proceed. Proof-of-concept experimental results have already been presented for electrochemically active microbes, such as *Geobacter sulfurreducens* [15, 16], *Shewanella oneidensis* MR-1 [17, 18], and *K. pneumoniae* L17 [19]. The production of biochemicals and biofuels with the bioelectrochemical system has also been investigated, e.g., butyrate production with *Clostridium tyrobutyricum* by instigating a cathodic reduction reaction [20] and the production of electron-dense metabolites (such as butanol and 1,3-propanediol) by *C. pasteurianum* [21]. Nevertheless, most of these studies focused on cathode-based electro-fermentation rather than an anode-based process. For anode-based studies, the production of ethanol and acetate by engineered *E. coli* and the production of ethanol by a co-culture of *G. sulfurreducens* and *C. cellobioparum* have been attempted; however, the metabolite titers were neither reported nor enhanced significantly compared to those in the conventional, non-bioelectrochemical system [22, 23].

In this study, anodic electro-fermentation by recombinant *K. pneumoniae* L17 was investigated for the production of 3-HP from glycerol (see the Additional file 1: Figure S1). This paper reports that the conventional limitation of the redox imbalance in fermentation can be overcome using bioelectrochemical system approaches. The results show that overexpressed AldH and the use of an electrode is well coordinated via a microbial anaerobic respiratory module. To the best of the authors’ knowledge, this is the first report of the successful application of the anodic electro-fermentation, and an improvement of 3-HP production.

## Methods

### Bacterial strains and media composition

To develop efficient 3-HP producer strains, *K. pneumoniae* L17 (herein after L17W) and *E. coli* DH5ɑ were purchased from CCTCC (China Center for Type Culture Collection) and KCCM (Korean Culture Center of Microorganisms), respectively. The recombinant *K. pneumoniae* strain was prepared using the pUC19/KGSADH vector, which is well-developed for high rates of expression of aldehyde dehydrogenase (AldH), with KGSADH (*ɑ*-ketoglutaric semialdehyde dehydrogenase) [10]. The transformation of pUC19/KGSADH to *K. pneumoniae* L17 (herein after L17K) was conducted using the method reported elsewhere [24]. Additional file 1: Table S1 lists the bacterial strains and plasmids used in this study (Additional file 1: Table S1). For strain maintenance, all strains used in this study were grown in LB medium and stored at −80 °C. The modified M9 medium was used for the 3-HP fermentation experiments using the following composition (per liter): 1 g NaCl, 1 g NH_4_Cl, 0.25 g MgSO_4_·7H_2_O, 13.53 g K_2_HPO_4_, 3.03 g KH_2_PO_4_, 1 g yeast extract, 2 g glucose, 11.05 g glycerol, 12.5 ml vitamin solution [25], and 12.5 ml trace element solution [25]. The medium was adjusted to pH 7.2 using 10% HCl and 5 N NaOH solutions. A 0.1 mM solution of filter-sterilized HNQ (2-hydroxy-1,4-naphthoquinone, neutral orange 6) was added to the anode chamber as an electron shuttle between the bacteria and electrode.

### Bioelectrochemical system (BES) configuration

H-type BES reactors were constructed from two glass bottles (310 ml capacity each, Duran, USA) with a glass tube bridge (inner diameter: 1.25 cm) containing a proton exchange membrane (PEM, Nafion 117, Dupont, Del. USA). A 20 wt% wet-proof carbon cloth (Nara Cell-Tech Co., Korea) was used for both the anode and cathode electrodes (2 cm × 5 cm). A Ag/AgCl electrode was installed in the anode chamber as the reference electrode. The anode chamber was filled with 250 mL of modified M9 medium. A 100 mM potassium ferricyanide solution dissolved in a 100 mM potassium phosphate solution (pH = 7.5) was added to the cathode chamber. All the components were pre-washed with 10% of HCl and 5 N NaOH solutions. The medium and BES reactors were sterilized at 121 °C for 15 min by autoclaving. Before starting the experiments, all strains tested were pre-cultured overnight in LB medium, and incubated for 12 h in M9 medium for activation. The pre-cultivated cells were inoculated in the anode chamber to an initial cell concentration of OD_600_ = 0.05. A 100 μM solution of ampicillin (L17W) or 50 μM of kanamycin (L17K) was added to the anode and cathode chamber to prevent contamination and plasmid stabilization.

### BES operation

Both the anode and cathode electrodes were connected via a 1 kΩ external resistance or a potentiostat for closed circuit operation (BES operating condition), whereas in non-BES operation, the anode was not connected to the resistance and cathode electrode (i.e., open circuit). Hence, the anode electrode in non-BES reactors does not accept the respiratory electrons produced from the metabolic pathway. Both BES and non-BES reactors were placed in a shaking incubator at 37 °C at 100 rpm. Before starting the BES experiments, both the anode and cathode chambers were purged with 99.9% of nitrogen gas to remove the dissolved oxygen. In the recombinant strain experiments, 0.2 mM of IPTG was added to induce AldH expression. The poised potential was applied to the anode using chronoamperometry (+0.5 V vs. Ag/AgCl) and a potentiostat (VersaSTAT 3, AMETEK, USA). The average current was monitored at 1 min intervals during the BES operation for glycerol fermentation.

### Inhibitor experiments

To determine the electron transfer mechanism between the anode electrode and bacterial anaerobic respiration for 3-HP production, the BES reactors were operated under spontaneous electron discharge to the anode with a fixed resistance (1 kΩ), instead of using a potentiostat. All the operating conditions were the same as those of the BES reactors described previously. The effect of the respiratory inhibitors such as rotenone (1 mM in final), antimycin A (0.5 μM), sodium azide (1 mM) were investigated in the BES reactor. The control BES reactor contained the same amount of ethanol and DI water, and was connected to a fixed resistance (1 kΩ). The current generation and 3-HP production with the different inhibitors were monitored.

### Analyses

Cell growth, pH change, NADH/NAD^+^ ratio, and metabolites production were determined by sampling the fermentation broth from the anode chamber at 0, 3, 6, 8, 12, 24, and 32 h. Planktonic cell growth and pH were determined by measuring the optical density (600 nm wavelength) and using a pH meter, respectively. The liquid samples were centrifuged at 5000 rpm for 10 min. The NADH/NAD^+^ ratio of the pellets was analyzed by colorimetric kit quantification (Biovision, USA), as described previously [9]. The supernatants were filtered using a syringe filter (0.22 μm, Nylon membrane, Whatman, UK). The filtered samples were analyzed to identify the metabolite concentrations by high performance liquid chromatography (HPLC, HP 1160 series, Agilent Technologies, USA) equipped with a 300 × 7.8 mm Aminex HPX-87H (Bio-Rad, USA) column at 65 °C and a refractive index (RI) and photodiode array (PDA) detector, using 2.5 mM H_2_SO_4_ as the mobile phase (flow rate = 0.5 mL/s). The currents generated were analyzed by a computer-based data acquisition (DAQ) system (NI-USB6218; National Instruments, USA) with LabVIEW™ (National Instruments, USA).

## Results and discussion

### Electron transfer to the anode electrode by L17W and L17K

**Fig. 2:**
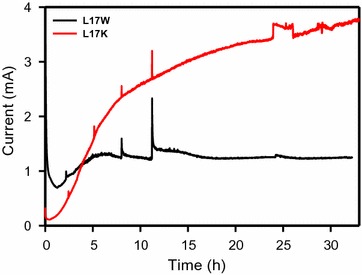
Current generation by L17W (*black*) and L17K (*red*) in BES. Anode was polarized to +0.5 V vs. Ag/AgCl

The anode electrode in the BES captures the respiratory electrons discharged from glycerol degradation, and the current (i.e., coulomb per sec, C/s) can be used as an indicator of the rate of extracellular electron transport from the strains. Figure 2 shows the current generation observed from the wild-type (L17W) and recombinant *K. pneumoniae* (L17K) in the BES reactors. Compared to L17W, L17K produced a significantly higher current under BES conditions. One hour after inoculation, the current generated increased exponentially and was correlated with cell growth (Additional file 1: Figure S2). Approximately 25.2 and 134.3 mmol of electrons were estimated to have been transferred to the electrode in the BES reactors by L17W and L17K, respectively, based on the estimation with Faraday’s constant (96,485 C/mole of e^−^). These results suggest that approximately 5 times more respiratory electrons were transferred from the L17K cell metabolism than that from L17W. The enhanced extracellular electron transfer to the electrode is expected to alter the metabolite production profile, along with the cellular redox rebalance.

### Glycerol consumption and production of metabolites in BES

**Fig. 3:**
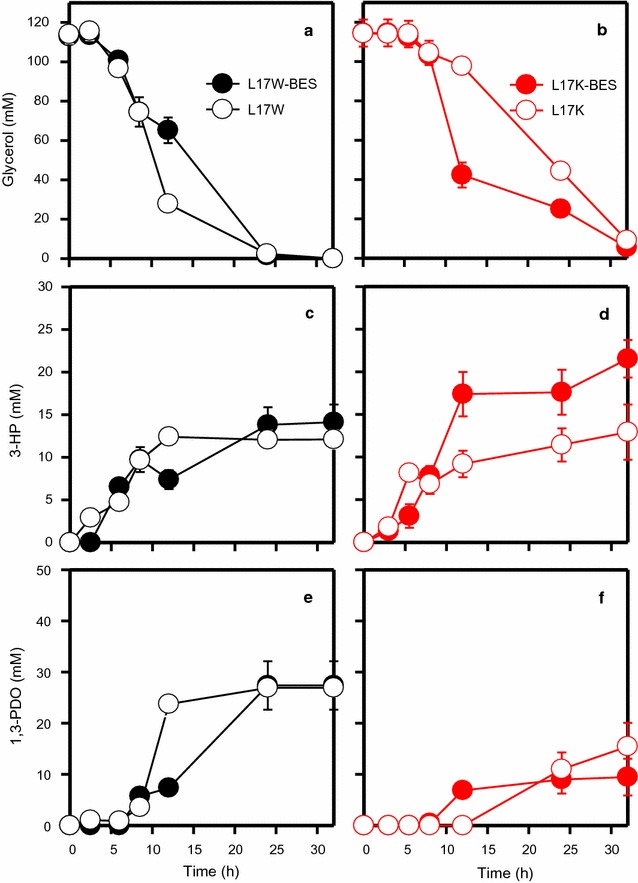
Time course profiles of glycerol consumption in L17W (**a**) and L17K (**b**), 3-HP production in L17W (**c**) and L17K (**d**) and 1,3-PDO production in L17W (**e**) and L17K (**f**)

The L17W and L17K strains were grown on glycerol in BES and non-BES, and cell growth and metabolite production were compared (Fig. 3 and Additional file 1: Figure S2). Under both conditions, L17W grew slightly better than L17K (Additional file 1: Figure S2). Nevertheless, the effects of electron flow to the electrode did not affect cell growth significantly under BES and non-BES circumstances. Figure 3 presents the concentration profiles of glycerol, 3-HP, and 1,3-propanediol (1,3-PDO) during fermentation. Most of the glycerol was consumed within 24 h when L17W was used, whereas 21 and 38% of the residual glycerol still remained with L17K fermentation: BES (25.0 ± 3.8 mM) and non-BES (44.3 ± 1.3 mM), respectively. The synthesis of 3-HP by L17W between BES and non-BES was similar (14.1 ± 2.1 and 12.0 ± 1.1 mM, respectively). Consequently, L17W produced a similar amount of 3-HP in both BES (14.1 ± 2.1 mM) and non-BES (12.0 ± 1.1 mM). In comparison, L17K produced 1.7 times more 3-HP in BES (21.5 ± 2.2 mM) than in non-BES (12.9 ± 3.2 mM).

**Fig. 4:**
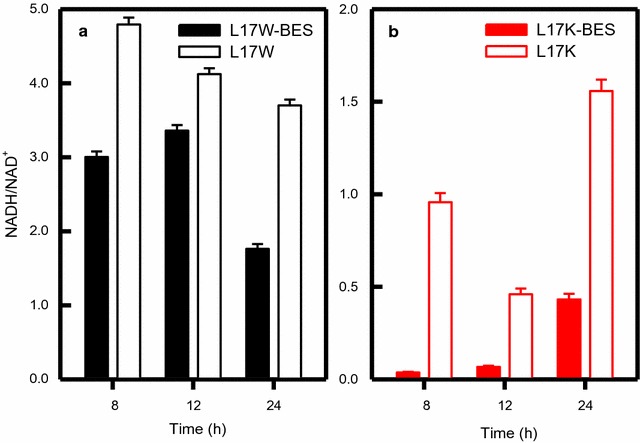
NADH/NAD^+^ ratio of L17W in BES and non-BES (**a**) and L17K in BES and non-BES (**b**)

The overexpression of AldH is essential to shift the metabolic flux of 3-hydroxypropanal (3-HPA) toward 3-HP from 1,3-PDO (Table 1). In recombinant L17K, anodic electro-fermentation appeared to have enhanced the directional selectivity to 3-HP between the two metabolic pathways (see Fig. 1). With L17W, the final 1,3-PDO production in BES and non-BES were similar: 27.4 ± 4.7 and 26.9 ± 1.7 mM, respectively. On the other hand, AldH-overexpressing L17K produced a lower 1,3-PDO in BES (9.46 ± 3.6 mM) than in non-BES (15.4 ± 4.6 mM). The increased 2,3-butanediol (2,3-BDO) production of L17K was observed only in BES (19.9 ± 0.3 mM), but not under the other conditions: 6.8 ± 0.1 and 6.7 ± 0.4 mM by L17W in BES and non-BES, respectively, 10.5 ± 2.0 mM by L17K in non-BES (Additional file 1: Figure S3). The production of other metabolites was similar in BES and non-BES (Additional file 1: Figure S3). To identify the cellular redox states, the NADH/NAD^+^ ratios were also determined (Fig. 4). BES with L17W showed a slightly lower ratio than that with non-BES (Fig. 4a). On the other hand, L17K exhibited a significantly lower NADH in BES than in non-BES (Fig. 4b), indicating that anodic electron transfer was quite high under the BES as shown in Fig. 2.

**Table 1:** Carbon and electron distributions of L17 under different operating conditions

Strains	Condition	Transferred electron to electrode (mmol)	3-HP (mM)	1,3-PDO (mM)	Carbon balance	Glycerol to 3-HPA^a^	3-HPA to 3-HP^b^
L17W	BES	25.2	14.1 ± 2.1	27.4 ± 4.7	0.81	0.37	0.34
	Non-BES	–	12.1 ± 1.1	26.9 ± 1.7	0.83	0.35	0.31
L17K	BES	134.3	21.5 ± 2.2	9.5 ± 3.6	0.79	0.27	0.69
	Non-BES	–	12.9 ± 3.2	15.4 ± 4.6	0.89	0.25	0.46

^a^(3-HP + 1,3-PDO)/glycerol consumption

^b^3-HP/(3-HP + 1,3-PDO)

### Shift of the cellular redox balance under BES conditions

The addition of HNQ to the culture medium can enhance the quinone shuttle-based electron transfer to the electrode, and activate the anaerobic respiratory pathways of *K. pneumoniae* L17 [19]. The metabolic flux under anaerobic conditions proceeds in a similar manner to that under (micro) aerobic conditions. Similar results were obtained in *Shewanella* sp. under MFC conditions, as reported by Matsuda et al. [26]. The introduction of AldH to *K. pneumoniae* increases electron transfer to the electrode significantly, as shown in Fig. 2. In a previous report, the *K. pneumoniae* DSMZ overexpressing AldH reduced more nitrate than the wild-type counterpart [9]. The improvement of anaerobic respiration (via nitrate reduction) was attributed to the coordination of NAD^+^-dependent oxidase (i.e., AldH) and NADH oxidoreductase (i.e., NADH:Ubiquinone reductase).

The glycerol metabolism and its carbon distribution in *K. pneumoniae* was determined by two regulons, glp and dha (see Fig. 1). The glp regulon includes glycerol kinase (GlpK) and glycerol-3-phosphate dehydrogenase (GlpD), both of which are expressed under aerobic conditions and play a major role in the oxidative assimilation of glycerol. The glp regulon can also be activated under anaerobic respiratory conditions, particularly with an extracellular electron acceptor. The GlpD enzyme is located in the cytoplasmic membrane and catalyzes the following reaction:

$${\text{Glycerol-3-phosphate }} + {\text{ quinone}} \to {\text{dihydroxyacetone-phosphate }} + {\text{ Quinol}}$$

In the other glycerol assimilation pathways, the dha modules consist of two reaction pathways involved in two associated enzymes, glycerol dehydrogenase (DhaD) with dihydroxyacetone kinase (DhaK), and glycerol dehydratase (DhaB) with propanediol oxidoreductase (DhaT). In the glycerol-reductive pathway catalyzed by DhaB, 3-HPA can be converted to either 3-HP or 1,3-PDO. The production of these two metabolites is conducted by controversial reactions, 3-HPA reduction for 1,3-PDO production (NADH-dependent propanediol dehydrogenase, dhaT) or 3-HPA oxidation for 3-HP production (NAD^+^-dependent aldehyde dehydrogenase, puuC). Therefore, the 1,3-PDO: 3-HP ratio was governed by the cellular redox states. The glycerol-reductive pathways are more highly activated under anaerobic conditions (i.e., high NADH/NAD^+^ ratio conditions); therefore, most of the 3-HPA would be transformed to 1,3-PDO in wild-type *K. pneumoniae*. In balanced fermentation (i.e., conventional fermentation), predominant 3-HP production from glycerol is practically difficult; therefore, co-production with 1,3-PDO was examined to simultaneously improve the titer and/or yield of 3-HP production by maintaining the cellular redox potential [27–30]. In this study, the electrode-based control of the cellular redox potential was applied to promote unbalanced fermentation with a decreased intracellular NADH level. These results suggest that the poised potential (+0.5 V vs. Ag/AgCl) can be applied to bioproduction not only for 3-HP biosynthesis, but also for the production of various biochemicals, which are required to overcome the thermodynamic barriers by changing the intracellular NADH/NAD^+^ level.

### Effects of the respiratory inhibitors on current generation and 3-HP production

**Fig. 5:**
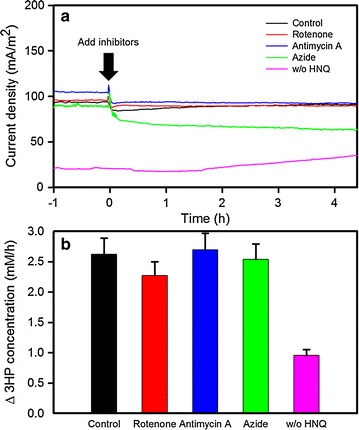
Current generation (**a**) and 3-HP production (**b**) with the respiratory inhibitors during BES operation. All BES reactors were operated under the condition of spontaneous electron discharge to the anode with a fixed resistance (1 kΩ). Control contained the same amount of ethanol and DI water instead of inhibitors

The coordination mechanism for the overexpressed AldH and anode electrode, was examined using various uncouplers of the membrane-bound electron transport protein. Spontaneous current generation was monitored in the presence of several respiratory inhibitors, such as rotenone (inhibitor of NADH dehydrogenase I), antimycin A (cytochrome c oxidoreductase), and azide (cytochrome oxidase). No significant decrease in current generation and 3-HP production were observed with the addition of rotenone and antimycin A (Fig. 5), while a relatively larger decrease in current was obtained with azide. Azide blocks electron flow between the cytochrome oxidase complex and terminal electron acceptors, such as oxygen and ferric compounds [31]. As a consequence, azide has been used to decrease the direct electron transport from the cell to the electrode in BES.

On the other hand, significantly less current and concurrently lower 3-HP production were obtained without HNQ, a quinone analogue (Fig. 5). This suggests that the electron shuttle (HNQ) pumps out the respiratory electron from putative quinone reductase on the cytoplasmic membrane, so that a lower NADH/NAD^+^ ratio can be maintained within the cell. These results suggest that shuttle-based electron transport is more dominant for 3-HP production in BES than direct electron transport. The NADH dehydrogenase II complex might act as an electron carrier between aldehyde dehydrogenase and the quinone pool, but it was not inhibited by the inhibitors tested.

### BES-based electron transport chain for 3-HP production

To implement a BES-based cellular redox control strategy into a bioprocess, an understanding of the electron transfer mechanisms and the effects of an externally poised potential on the production rate (3-HP in this study) are needed. Although previous studies revealed various spontaneous electron transfer modules in exoelectrogens, the mechanisms of quinone-based electron transfer have not been examined extensively. In the present study, it was hypothesized that the externally added quinone derivative mediator (HNQ) might interact with the respiratory enzymes, e.g., NADH dehydrogenase complex I and quinone:cytochrome c oxidoreductase. Interestingly, no significant difference in current generation and 3-HP production rate were observed in the presence of rotenone and antimycin A, which are inhibitors used widely for NADH dehydrogenase complex I and quinone:cytochrome c oxidoreductase, respectively (Fig. 5).

**Fig. 6:**
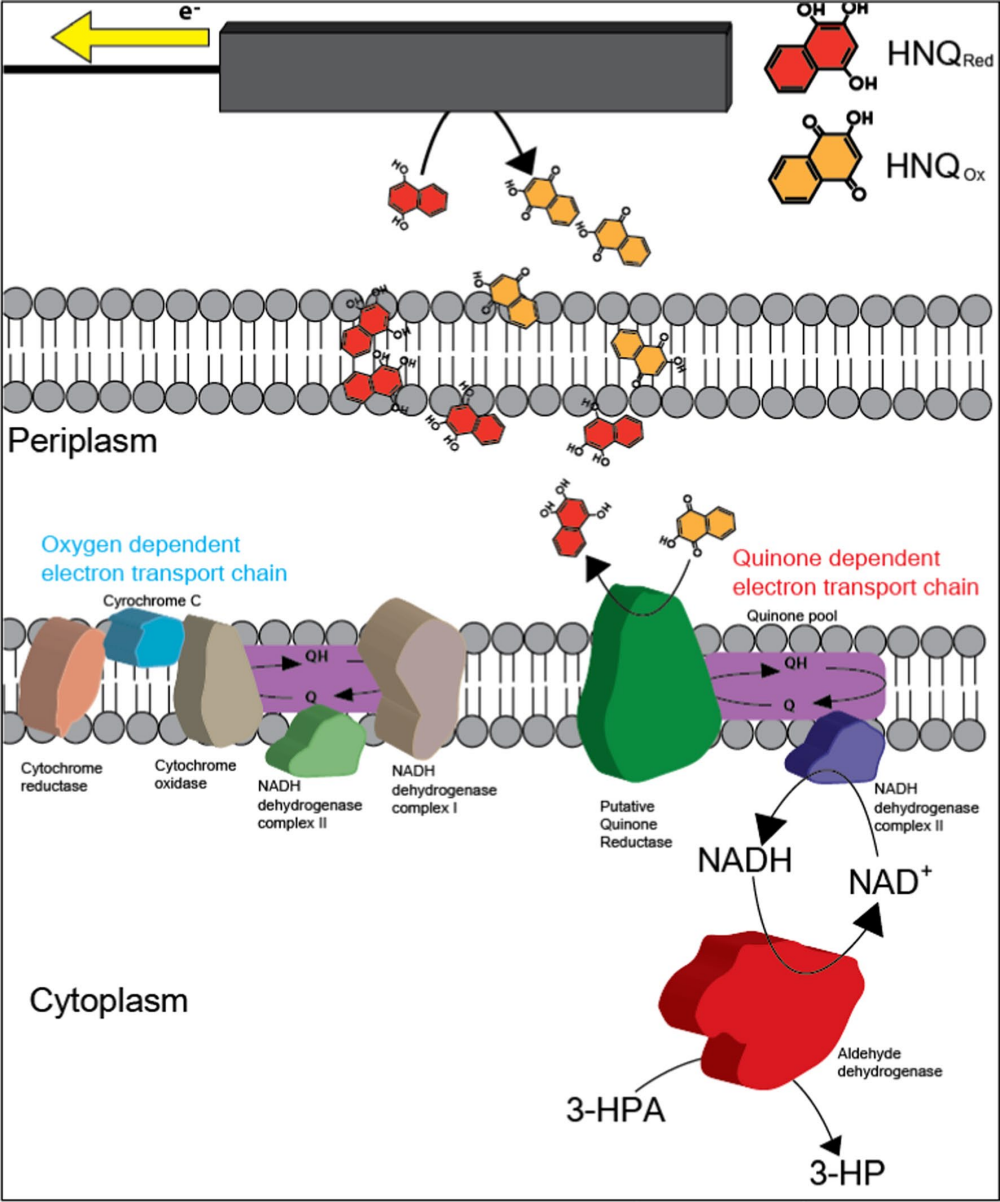
Hypothetical mechanism for the interaction between AldH and the electrode during 3-HP production in BES

Rothery et al. reported that naphthoquinone can be reduced easily by various anaerobic quinone reductases, such as menaquinol–nitrate oxidoreductase and menaquinol–fumarate oxidoreductase [32]. The present results with ETC inhibitors suggest that a NADH:quinone oxidoreductase (probably NADH dehydrogenase complex II) and quinone reductase might be activated during the anaerobic respiration of *K. pneumoniae* in BES. Electron flow during glycerol conversion to 3-HP with *K. pneumoniae* in BES can be hypothesized as follows: (1) NADH formed by KGSADH is oxidized by a putative NADH:quinone oxidoreductase (or NADH dehydrogenase complex II), (2) reduced menaquinones (in the quinone pool) deliver their electrons to anaerobic quinone reductase(s), (3) HNQ reduction occurs by the action of the putative anaerobic quinone reductase(s), and (4) reduced HNQ transfers electrons to the electrode (Fig. 6). Interestingly, the AldH overexpressed *K. pneumoniae* (L17K) produced higher 3-HP levels in BES, while the wild-type strain (L17W) both in BES and non-BES, and L17K in non-BES, showed similar levels of 3-HP production (Fig. 3). These results suggest that AldH interacts indirectly with the electrode via HNQ to deliver a respiratory electron. Further study for a synergistic pathway for combined metabolic engineering and BES will provide a platform technology to regulate the cellular redox potential, and improve the yield and titer of the target value-added chemicals.

The substitution of a petroleum-based chemical refinery with a sustainable biorefinery has been highlighted as one of the future goals of green technology. With the help of the recent advances in multi-OMICS and innovative bioprocesses, the biological production of various platform chemicals and an improvement of their yield/titer have become easier. Nevertheless, regulation and control of the cellular redox potential to improve the yield/selectivity and reduce the production cost, remains a challenge. Electro-fermentation is a promising alternative to traditional redox control methods, such as oxygen sparging, and the addition of co-substrates and chemical reagents [33]. Furthermore, electro-fermentation can provide a tool to break the theoretical maximum productivity of conventional fermentation, which is set by the innate cellular metabolism. The strategy suggested in the present study can be also used as an energy storage and efficient utilization method for electrical energy from diverse electricity-based renewable energy and a carbon-neutral power plant with the simultaneous production of high value-added chemicals [34].

This paper is the first report on the enhancement of 3-HP bioproduction using electro-fermentation. The BES-based cellular redox control strategy demonstrated proof-of-principle, even though the 3-HP titer and volumetric productivity so far lack the levels for industrial relevance. To achieve industrial-scale application, further study will be needed to develop a large-scale and cost-effective BES reactor for electro-fermentation, by implementing electrode-based anodic and cathodic reactors. The membrane-based BES processes (e.g., electrodialysis) which proceed the production and separation of products simultaneously, have been extensively investigated, and are highly applicable to electro-fermentation [35–38]. The finely tunable electrochemical control strategy as well as genetic and metabolic engineering to redirect the metabolic fluxes and remove the byproduct production pathways, are also anticipated.

## Conclusions

The application of a bioelectrochemical system facilitated the enhanced production of 3-HP via the activation of anaerobic respiration to regulate the NADH/NAD^+^ ratio. The overexpressed AldH and electrode-based respiration mediated by an electron shuttle resulted in higher 3-HP production under BES conditions. This study provides a novel strategy to control the cellular redox states to overcome the yield and titer limited in conventional fermentation, which can be applied not only to 3-HP production, but also to other bioconversion processes through altered cell redox states.

## Additional file

**Additional file 1.** Additional figures and table. **Table S1**. Strains and plasmid used in this study. **Fig. S1**. Cell growth profile of L17W in BES and non-BES (A) and L17K in BES and non-BES (B) and pHprofile of L17W in BES and non-BES (C) and L17K in BES and non-BES (D). **Fig. S2**. Metabolites profile of L17W (A) and L17K (B) in BES and L17W (C) and L17K (D) in non-BES.

## References

[1] Della Pina C, Falletta E, Rossi M (2011). A green approach to chemical building blocks. The case of 3-hydroxypropanoic acid. Green Chem.

[2] Kumar V, Ashok S, Park S (2013). Recent advances in biological production of 3-hydroxypropionic acid. Biotechnol Adv.

[3] Werpy T, Petersen G, Aden A, Bozell J, Holladay J, White J, Manheim A, Eliot D, Lasure L, Jones S (2004). Top value added chemicals from biomass.

[4] Jung WS, Kang JH, Chu HS, Choi IS, Cho KM (2014). Elevated production of 3-hydroxypropionic acid by metabolic engineering of the glycerol metabolism in *Escherichia coli*. Metab Eng.

[5] Li Y, Wang X, Ge X, Tian P (2016). High production of 3-hydroxypropionic acid in *Klebsiella pneumoniae* by systematic optimization of glycerol metabolism. Sci Rep.

[6] Lim HG, Noh MH, Jeong JH, Park S, Jung GY (2016). Optimum rebalancing of the 3-hydroxypropionic acid production pathway from glycerol in *Escherichia coli*. ACS Synth Biol.

[7] Sankaranarayanan M, Ashok S, Park S (2014). Production of 3-hydroxypropionic acid from glycerol by acid tolerant *Escherichia coli*. J Ind Microbiol Biotechnol.

[8] Jiang X, Meng X, Xian M (2009). Biosynthetic pathways for 3-hydroxypropionic acid production. Appl Microbiol Biotechnol.

[9] Ashok S, Raj SM, Ko Y, Sankaranarayanan M, Zhou S, Kumar V, Park S (2013). Effect of puuC overexpression and nitrate addition on glycerol metabolism and anaerobic 3-hydroxypropionic acid production in recombinant *Klebsiella pneumoniae* ΔglpKΔdhaT. Metab Eng.

[10] Ko Y, Ashok S, Zhou S, Kumar V, Park S (2012). Aldehyde dehydrogenase activity is important to the production of 3-hydroxypropionic acid from glycerol by recombinant *Klebsiella pneumoniae*. Process Biochem.

[11] Keuth S, Bisping B (1994). Vitamin B12 production by *Citrobacter freundii* or *Klebsiella pneumoniae* during tempeh fermentation and proof of enterotoxin absence by PCR. Appl Environ Microbiol.

[12] Ye K, Shijo M, Jin S, Shimizu K (1996). Efficient production of vitamin B12 from propionic acid bacteria under periodic variation of dissolved oxygen concentration. J Ferment Bioeng.

[13] Gonzalez P, Correia C, Moura I, Brondino C, Moura J (2006). Bacterial nitrate reductases: molecular and biological aspects of nitrate reduction. J Inorg Biochem.

[14] Lovley DR, Nevin KP (2011). A shift in the current: new applications and concepts for microbe-electrode electron exchange. Curr Opin Biotechnol.

[15] Segura D, Mahadevan R, Juárez K, Lovley DR (2008). Computational and experimental analysis of redundancy in the central metabolism of *Geobacter sulfurreducens*. PLoS Comput Biol.

[16] Yang TH, Coppi MV, Lovley DR, Sun J (2010). Metabolic response of *Geobacter sulfurreducens* towards electron donor/acceptor variation. Microb Cell Fact.

[17] Biffinger JC, Ray R, Little BJ, Fitzgerald LA, Ribbens M, Finkel SE, Ringeisen BR (2009). Simultaneous analysis of physiological and electrical output changes in an operating microbial fuel cell with *Shewanella oneidensis*. Biotechnol Bioeng.

[18] Tang YJ, Meadows AL, Kirby J, Keasling JD (2007). Anaerobic central metabolic pathways in *Shewanella oneidensis* MR-1 reinterpreted in the light of isotopic metabolite labeling. J Bacteriol.

[19] Kim C, Ainala SK, Oh Y-K, Jeon B-H, Park S, Kim JR (2016). Metabolic flux change in *Klebsiella pneumoniae* L17 by anaerobic respiration in microbial fuel cell. Biotechnol Bioprocess Eng.

[20] Choi C, Cui Y (2012). Recovery of silver from wastewater coupled with power generation using a microbial fuel cell. Bioresour Technol.

[21] Choi O, Kim T, Woo HM, Um Y (2014). Electricity-driven metabolic shift through direct electron uptake by electroactive heterotroph *Clostridium pasteurianum*. Sci Rep.

[22] Speers AM, Young JM, Reguera G (2014). Fermentation of glycerol into ethanol in a microbial electrolysis cell driven by a customized consortium. Environ Sci Technol.

[23] Sturm-Richter K, Golitsch F, Sturm G, Kipf E, Dittrich A, Beblawy S, Kerzenmacher S, Gescher J (2015). Unbalanced fermentation of glycerol in *Escherichia coli* via heterologous production of an electron transport chain and electrode interaction in microbial electrochemical cells. Bioresour Technol.

[24] Fournet-Fayard S, Joly B, Forestier C (1995). Transformation of wild type *Klebsiella pneumoniae* with plasmid DNA by electroporation. J Microbiol Methods.

[25] Kim JR, Premier GC, Hawkes FR, Rodríguez J, Dinsdale RM, Guwy AJ (2010). Modular tubular microbial fuel cells for energy recovery during sucrose wastewater treatment at low organic loading rate. Bioresour Technol.

[26] Matsuda S, Liu H, Kouzuma A, Watanabe K, Hashimoto K, Nakanishi S (2013). Electrochemical gating of tricarboxylic acid cycle in electricity-producing bacterial cells of *Shewanella*. PLoS ONE.

[27] Ashok S, Raj SM, Rathnasingh C, Park S (2011). Development of recombinant *Klebsiella pneumoniae* ∆dhaT strain for the co-production of 3-hydroxypropionic acid and 1, 3-propanediol from glycerol. Appl Microbiol Biotechnol.

[28] Huang Y, Li Z, Shimizu K, Ye Q (2012). Simultaneous production of 3-hydroxypropionic acid and 1, 3-propanediol from glycerol by a recombinant strain of *Klebsiella pneumoniae*. Bioresour Technol.

[29] Huang Y, Li Z, Shimizu K, Ye Q (2013). Co-production of 3-hydroxypropionic acid and 1, 3-propanediol by *Klebseilla pneumoniae* expressing aldH under microaerobic conditions. Bioresour Technol.

[30] Kumar V, Sankaranarayanan M, Jae KE, Durgapal M, Ashok S, Ko Y, Sarkar R, Park S (2012). Co-production of 3-hydroxypropionic acid and 1, 3-propanediol from glycerol using resting cells of recombinant *Klebsiella pneumoniae* J2B strain overexpressing aldehyde dehydrogenase. Appl Microbiol Biotechnol.

[31] Elbehti A, Brasseur G, Lemesle-Meunier D (2000). First evidence for existence of an uphill electron transfer through the bc1 and NADH-Q oxidoreductase complexes of the acidophilic obligate chemolithotrophic ferrous ion-oxidizing bacterium *Thiobacillus ferrooxidans*. J Bacteriol.

[32] Rothery RA, Chatterjee I, Kiema G, Mcdermott MT, Weiner JH (1998). Hydroxylated naphthoquinones as substrates for *Escherichia coli* anaerobic reductases. Biochem J.

[33] Schievano A, Sciarria TP, Vanbroekhoven K, De Wever H, Puig S, Andersen SJ, Rabaey K, Pant D (2016). Electro-fermentation-merging electrochemistry with fermentation in industrial applications. Trends Biotechnol.

[34] Moscoviz R, Toledo-Alarcón J, Trably E, Bernet N (2016). Electro-fermentation: how to drive fermentation using electrochemical systems. Trends Biotechnol.

[35] Zhang Y, Olias LG, Kongjan P, Angelidaki I (2011). Submersible microbial fuel cell for electricity production from sewage sludge. Water Sci Technol.

[36] Zhang Y, Angelidaki I (2015). Bioelectrochemical recovery of waste-derived volatile fatty acids and production of hydrogen and alkali. Water Res.

[37] Kim C, Lee CR, Song YE, Heo J, Choi SM, Lim D-H, Cho J, Park C, Jang M, Kim JR (2017). Hexavalent chromium as a cathodic electron acceptor in a bipolar membrane microbial fuel cell with the simultaneous treatment of electroplating wastewater. Chem Eng J.

[38] Gildemyn S, Verbeeck K, Slabbinck R, Andersen SJ, Prévoteau A, Rabaey K (2015). Integrated production, extraction, and concentration of acetic acid from CO_2_ through microbial electrosynthesis. Environ Sci Technol Lett.

