# Supplementary material for: Anodic electro-fermentation of 3-hydroxypropionic acid from glycerol by recombinant Klebsiella pneumoniae L17 in a bioelectrochemical system
Source: Biotechnol Biofuels. 2017 Aug 17;10:199. doi: 10.1186/s13068-017-0886-x (PMC5561608; doi:10.1186/s13068-017-0886-x)
Supplement: Supplementary file 1 — Additional file 1. Additional figures and table. Table S1. Strains and plasmid used in this study. Fig. S1. Cell growth profile of L17W in BES and non-BES (A) and L17K in BES and non-BES (B) and pHprofile of L17W in BES and non-BES (C) and L17K in BES and non-BES (D). Fig. S2. Metabolites profile of L17W (A) and L17K (B) in BES and L17W (C) and L17K (D) in non-BES. [file 13068_2017_886_MOESM1_ESM.docx]

**Supplementary Information**

**Anodic electro-fermentation of 3-hydroxypropionic acid from glycerol by recombinant *Klebsiella pneumoniae* L17 in bioelectrochemical system**

**Changman Kim, Mi Yeon Kim, Iain Michie, Byong-Hun Jeon, Giuliano C. Premier, Sunghoon Park, Jung Rae Kim^†^**

^†^**Corresponding author:**

Jung Rae Kim, Ph.D

*Address*: School of chemical and biomolecular engineering, Pusan National University, Busan 609-735, Republic of Korea

*E-mail address*: [j.kim@pusan.ac.kr](mailto:j.kim@pusan.ac.kr)

*Phone*: +82.51.510.2393

*Fax*: +82.51.510.3943

**Table S1**. Strains and plasmid used in this study

**Fig. S1**. Cell growth profile of L17W in BES and non-BES (A) and L17K in BES and non-BES (B) and pH profile of L17W in BES and non-BES (C) and L17K in BES and non-BES (D)

**Fig. S2**. Metabolites profile of L17W (A) and L17K (B) in BES and L17W (C) and L17K (D) in non-BES

Table S1. Strains and plasmid used in this study

| Strain and plasmid | Description | Source |
| --- | --- | --- |
| **Strains** |  |  |
| *E. coli* DH5ɑ | Cloning host | KCCM, Korea |
| L17W | Expression host (*Klebsiella pneumoniae* L17) | CCTCC, China |
| L17K | *Klebsiella pneumoniae* L17 harboring KGSADH on the pUC19 plasmid | In this study |
| **Plasmid** |  |  |
| pUC19/KGSADH | pUC19-lac promoter; KGSADH; Km^r^ | Ko et al[[11](#_ENREF_11)] |

Fig. S1. Cell growth profile of L17W in BES and non-BES (A) and L17K in BES and non-BES (B) and pH profile of L17W in BES and non-BES (C) and L17K in BES and non-BES (D)

Fig. S2. Metabolites profile of L17W (A) and L17K (B) in BES and L17W (C) and L17K (D) in non-BES
